# Winter Fads

**Published:** 1910-11-12

**Authors:** 


### WINTER FADS.

LUCRETIUS, who knew mankind probably as well as any great writer, somewhere in his monumental poem remarks that the seasons breed their various virtues in men's brains. The passage is one which at first sight suggests the Galenical theory of humoral pathology and the old-time faiths which we still reverence without comprehending their inward meaning, in these degenerate days when we speak of "lunatics" and the carotid artery. Every old writer in medicine has something to remark anent this influence of the seasons upon diseases, and the author of "De Rerum Natura" does not stand solitary in his declaration that summer brings liveliness and winter moodiness to normal men. Trolula of Salern, the wise daughter-in-law of old Copho, first of lady doctors (and if tradition speaks true, of militant suffragettes as well), ascribed the "bladder stone" to autumn wind and brain tumours to winter cold. Ambrose Parè, wiser than most of his generation of professional brethren, thought gastric diseases were induced by summer heat; and to come to more recent times, Boerhaave held that spring breezes were causative factors in fevers. The list may easily be extended, but it is unnecessary to do so. What we are here concerned with is the recrudescence of the tendency to attribute to the seasons certain inherent qualities, whether negative for good or positive for evil, which manifest themselves at times in strange and bizarre fashion.

The faddist, like the poor man, is always with us—nay, more, if by any blessed combination of sociological and economic forces we ever succeed in solving the problem of pauperism we shall still have among us those who firmly believe the wonderful gospel of red-lettered dietetics, whose sanctuary is a little restaurant not far removed from where we write these lines. And it would be well for mankind if our cranks were so little pernicious as the two classes we have specified; but there is unfortunately a vast and increasing crowd of irresponsibles who find in eccentricity an advertisement as well as a pastime, and enjoy the two consequences sometimes at the expense of their own bodily health. In summer the hygienic faddist cannot do any great amount of harm. Then the hatless brigade, as Dr. Dockrell is always reminding us, runs a risk of dirtying and making brittle its hair; and the sandalled family probably is all the better for its return to an aboriginal bootless state. But in winter the case is entirely different. Every family practitioner can tell tales, if he choose, of the mischief wrought by faddist parents who, with the intention of "hardening" their offspring, have exposed the latter to conditions directly detrimental to health. The cold bath, in which the ice has to be broken before it can be made ready for the hard-frozen sponge, is a fetish which is dying hard; the sooner it is extinct the better for the race. It serves no useful cleansing purpose, and in the vast majority of cases habitual submission to its torture is merely a striking example of what St. Augustine calls the "arrogant pride of the unknowing." Where its use is not followed by an immediate reaction it is positively dangerous, and a moderately unchilled bath on a winter's morning is far more beneficial and wholesome. Equally to be condemned, in the case of children, at least, is the practice of exposing the knees and lower parts of the thighs by wearing stockings that reach only half-way up the leg, and trousers or skirts that are so nicely graduated that their lower limit may well serve to mark the apex of Scarpa's triangle. It is true that this type of costume is popular in Scotland; but it is permissible to point out that even there it is a relic if not of barbarism, at any rate of the time when a Highland boy, to sleep warmly on a winter's night, dipped his plaid in water, wrapped it round his body, and awoke the next morning without the slightest need for a dose of aspirin. We no longer breed that race, either in the Highlands or elsewhere, and certainly not in crowded cities where the practice of the open knee is peculiarly popular. It need only be added that while such a practice may harden three per cent. of growing boys and girls it undoubtedly militates against the good health of ninety-seven per cent. It may be said that these are unnecessary warnings, likely to lead to the reverse, the mollycoddling extreme; but there is a sane mean between the two limits which will appeal to sensible folk. It is that mean which the practitioner should do his utmost to get the public to accept. He is not the arbiter of fads, but he is often consulted by the most rabid faddist, and a word in season can work wonders if it is supported by a simple explanation of principles.

